# Supplementary material for: Essential protein P116 extracts cholesterol and other indispensable lipids for Mycoplasmas
Source: Nat Struct Mol Biol. 2023 Feb 13;30(3):321–9. doi: 10.1038/s41594-023-00922-y (PMC10023570; doi:10.1038/s41594-023-00922-y)
Supplement: Supplementary file 1 — Supplementary Tables 1 and 2 and the captions and legends of the Supplementary Movies. [file 41594_2023_922_MOESM1_ESM.pdf]

nature structural & molecular biology

Article https://doi.org/10.1038/s41594-023-00922-y

Essential protein P116 extracts cholesterol and other indispensable lipids for *Mycoplasmas*

In the format provided by the authors and unedited

Supplementary Information

Supplementary Table I. Number of cell detachments and motility status in microcinematography (MCN) replicates of *M. pneumoniae* cells before and after adding polyclonal antibodies (PCA) vs P1 and P116. A negative control was performed by incubating cells in the same medium (PBS - 10% FBS) but no antibodies were added. Mean and standard deviation (SD) were computed from the frequencies of detached cells in each replicate.

		Before AS			After AS				
**PCA**	**MCN replicate**	**Total**	**Non-Motile**	**Motile**	**Total**	**Non-Motile**	**Motile**	**Detached cells**	**% Detached cells**
**P1**	**1**	46	5	41	15	11	4	37	90,2
	**2**	20	1	19	4	4	0	19	100,0
	**3**	131	3	128	31	24	7	121	94,5
	**4**	15	0	15	9	9	0	15	100,0
	**5**	397	9	388	122	89	33	355	91,5
								**Mean**	**95,1**
								**SD**	**4,6**
**P116**	**1**	170	5	165	112	7	105	60	36,4
	**2**	79	7	72	45	8	37	35	48,6
	**3**	51	5	46	35	5	30	16	34,8
	**4**	42	5	37	30	6	24	13	35,1
	**5**	61	4	57	31	4	27	30	52,6
								**Mean**	**40,2**
								**SD**	**6,6**
**Negative control**	**1**	29	4	25	12	4	8	17	68,0
	**2**	49	2	47	31	5	26	21	44,7
	**3**	32	4	28	17	4	13	15	53,6
								**Mean**	**53,8**
								**SD**	**11,8**

Supplementary Table II. Relative level of ApoAI in both HDL in the radioactive assay and P116 after incubating with HDL

	ApoAI (mg/mL)
**HDL**	
Free cholesterol-radiolabeled form	0.2272
Esterified cholesterol-radiolabeled form	0.2272
**P116 after incubating with HDL**	
Empty	0.00
Refilled	0.00

Synthetic DNA for P116

ATGAAACTGAGCGCGATTATTAGCCTGAGCGTTGCGGGCACCGTTGGCACCACCGCGGTTGTTGTTCCGACCACCATTACCCTGGTTAACAA
GACCCACCAGGTTGAGCACGAAAGCGAGCAGAGCGACTTTCAAGATATTCGTTTCGGTCTGAACAGCGTGAAGCTGCCGAAAGCGCAGCCG
GCTGCGGCGACCCGTATCACCGTTGAGAACGGCACCGATAAGCTGGTGAACTACAAAAGCAGCCCGCAGCAACTGTTCCTGGCGAAAAACG
CGCTGAAGGACAAACTGCAGGGCGAGTTTGACAAGTTCCTGAGCGATGCGAAAGCGTTTCCGGCGCTGACCGCGGACCTGCAGGAATGGG
TTGATCAGCAACTGTTCAACCCGAACCAGAGCTTCTTCGACCTGAGCGCGCCGCGTAGCAACTTTACCCTGAGCAGCGACAAGAAAGCGAG
CCTGGATTTCATTTTTCGTTTCACCAACTTCACCGAGAGCGTTCAACTGCTGAAGCTGCCGGAAGGCGTGAGCGTGGTTGTGGACAGCAAAC
AGAGCTTTGATTACTATGTTAACGCGAGCGCGCAAAAGCTGCTGGTGCTGCCGCTGAGCCTGCCGGACTACACCCTGGGTCTGAACTATAT
GTTCGATCACATCACCCTGAACGGCAAGGTTGTGAACAAATTTAGCTTCAACCCGTTTAAAACCAACCTGAACCTGGCGTTCAGCAACGTTT
ATAACGGTGTTGATGTGTTCGAGGCGCAGAAGAACCTGGTTGGCAAGGGCAAATACCTGAACACCCACGTGAAAGCGGAAGACGTTAAGA
AAGATGTGAACGCGAACATCAAGAACCAGTTTGACATTGCGAAAATCATTGCGGAGCTGATGGGCAAGGCGCTGAAAGAATTTGGTAACCA
GCAAGAGGGCCAACCGCTGAGCTTCCTGAAGGTTATGGACAAGGTGAAAGAAGATTTTGAGAAACTGTTCAACCTGGTTCGTCCGGGTCTG
GGCAAGTTCGTGAAGGACCTGATCCAGAGCAGCAGCCAAGCGGAAAACAAGATCACCGTGTACAAACTGATCTTCGACAACAAGAAAACC
ATCCTGAACCTGCTGAAGGAACTGAGCATTCCGGAGCTGAACAGCAGCCTGGGTCTGGTTGACGTGCTGTTCGATGGTATCACCGACAGCG
ATGGCCTGTATGAGCGTCTGCAGAGCTTTAAGGACCTGATTGTTCCGGCGGTGAAGACCAACGAAAAAACCGCGGCGCTGAGCCCGCTGA
TTGAGGAACTGCTGACCCAGAAAGACACCTACGTTTTCGATCTGATCCAAAAGCACAAAGGTATTCTGACCAACCTGCTGAAGAACTTTCTG
GCGGACTTCCAGAAAAGCACCCCGTTTATGGCGGATCAAGTGGCGATCTTTACCGAACTGTTCGACAACGAGGGTGCGTTTGACCTGTTCG
GCGAGGCGGACTTTGTTGATAAAATCGCGGAACTGTTCCTGACCAAGCGTACCGTGAAAAACGGCGAAAAGATTGAAACCAAAGACAGCCT
GCTGGTTACCAGCCTGAAGAGCCTGCTGGGCGAGAAAGTTGCGGCGCTGGGCGACCTGCTGGATAGCTATATCTTCAAGAACGAACTGCTG
AACCGTAGCGTTGAAGTGGCGAAAGCGGAGGCGAAGGACACCAAAGGTGCGACCGATTACAAGAAAGAGCAGGCGAAGGCGCTGAAGAA
ACTGTTCAAACACATCGGCGAAAACACCCTGAGCAAGACCAACCTGGATAAAATTACCCTGAAAGAGGTGAAAAACACCGAAAACGTGGAG
CTGGAGGAAACCGAAACCACCCTGAAGGTGAAGAAACTGGACGTTGAATATAAAGTGGAGCTGGGTAACTTTGAGATCAAGAACGGCCTG
ATTAAAGCGATGCTGGAGTTCCTGCCGGACACCAAGGATCTGGAAACCACCCTGGATAAGCTGCTGTTCAAAGGCGAGAGCTACAAGGCG
ATGAAGGACAAGTACATCAAAGAAGGCTTCCCGGGTTATGGTTGGGCGAAGGGTGTTGTGCCGGGTGCGTTTGAAAGCATCGAGAACACCT
TCAAAAGCGCGATCGATAAGACCAAAAGCATTCGTGACCTGTTTGGTGATATGCTGTTCGGCAACGACCTGAGCAGCGTTAAGGAAACCGA
TAGCTTTATCACCCTGGGTGGCAGCTTCGACATTAAATATGGTGGCGAGAACCTGAACGTTCTGCCGGCGTACTATAGCCTGATCAACAGCG
AAATTGGTTACCAGATCATTGGCGTGGACACCACCATTGATGCGACCAAGGTTAAAGTGGAACTGAAGAACAAAGAGTATAAGGGTAAAAG
CCCGGCGATCAACGGCCAGGTTAAACTGAGCCAGAGCTTCTTCAACGTGTGGACCAACATGTTCGACAGCATCACCAAGCAGATTTTTCAAA
AGAAATACGAGTTCAAGGACAACATTCAAGTGTTTGCGCGTAACGAAGATAACACCAGCCGTCTGGAGCTGGACATCAGCGATCCGGAAC
AGCGTGTTATTCCGTTTGCGTTCGTGGACGGTTTCGGCATCCAACTGAAGGCGGTTGATAAAAACATTACCAAGGAAGCGGGTAACACCGA
GCCGAAGAGCCCGGTGATCCAGCTGTATGAAGCGCTGAACAAGGAGAAAGACCAAAAACAGCAAAGCAAGCAGAGCCCGAAACAACTGGA
TACCAAGACCCAGCTGGGTTACCTGCTGAAACTGGGCGACAACTGGAGCAAGGACGATTATAAAAGCCTGATTGACGATACCATCATTAACA
ACAACTACCTGGAGGCGAGCTTCAACAGCAAAATCACCGTTGACCGTCTGGGTATCCCGATTGATCTGTGGCTGTTTAAAATCTGGCCGAAA
TTCAACCTGGAAATTCCGATGCAGGGTAGCCTGCAACTGTACAGCAGCAGCGTGATCTTCCCGTACGGCATTTATGACACCAGCGTTCAGGA
TGCGGCGAAGATCGTGAAACGTCTGAACTTTACCGACATGGGTTTTAAGCTGAACGATCCGAAGCCGAACTTTTGGTTTGTTGGTTTCTAA

Primers	Sequence 5’-3’
Fwd $P116_{30}$	**AGGAGATATACCATGAACAAGACCCACCAGGTTGAG**
Fwd $P116_{246}$	**AGGAGATATACCATGGGTGTTGATGTGTTCGAG**
Fwd $P116_{13}$	**AGGAGATATACCATGGGCACCGTTGGCACCACCGCG**
Rev $P116_{957}$	**GTGATGGTGATGTTTACCCAGACGGTCAACGGTGATTTG**
Rev $P116_{845}$	**GTGATGGTGATGTTTGCTGATGTCCAGCTCCAG**

Fwd P116_W_{681}A	TTCCCGGGTTATGGTGCGGCGAAGGGTGTTGTG
Rev P116_W_{681}A	'CACAACACCCTTCGCCGCACCATAACCCGGGAA

Supplementary Videos

Supplementary Video 1: Microcinematography adding P1 polyclonal antibodies. Frames were taken each 0.5 seconds of observation and are showed at 20 frames per second in the movie. The blank frame/s in the first seconds of the movie denote when the polyclonal antibodies were added to cells.

Supplementary Video 2: Microcinematography adding P1116 polyclonal antibodies. Frames were taken each 0.5 seconds of observation and are showed at 20 frames per second in the movie. The blank frame/s in the first seconds of the movie denote when the polyclonal antibodies were added to cells.

Supplementary Video 3: Control microcinematography. Frames were taken each 0.5 seconds of observation and are showed at 20 frames per second in the movie.

Supplementary Video 4: Density map of the P116 homodimer
Rotation along the long axis of the cryoEM density map. The complete extracellular region of the P116 dimer at 3.3 Å resolution, 90 degrees apart. The individual domains can be appreciated: The dimerization interface (shown in pink), the core domains with the four contiguous antiparallel helices (shown in blue) and a β-sheet with five antiparallel strands (shown in orange) as well as the N-terminal domain is shown in green (Color coding is identical to the coloring in the Figures 1 &2).

Supplementary Video 5: Ribbon model of P116

Ribbon representation of the P116 structure with the same coloring as in Movie 1.

Supplementary Video 6: Flexibility of P116

Animation of the wringing motion between the monomers as seen after classification of the cryoEM data. In some classes both cavities face the same direction, while in other they face 80 degrees apart. The speed of the movie may be adjusted in order to appreciate the conformational change. The N- terminal domains were computationally removed for the better visualization.

Supplementary Video 7: Hydrophobicity map of the P116 homodimer

Rotation along the long axis of the hydrophobic map of P116. The huge hydrophobic cavity that is fully accessible to solvent can be appreciated.

Supplementary Video 8: Crosssection of one P116 monomer

Cross-section of the hydrophobic map of the monomer reveals the positions of the ligands (in red).

Supplementary Video 9: Ribbon model of a P116 monomer (colors as in Figure 2 & 3) with the unaccounted elongated densities.

Ribbon representation of the monomer with the ligands (in red). The alignment of the ligands along the bridge helix can be seen.

Supplementary Video 10: Conformational change of P116 top view

Morphing between the ribbon models from P116 and P116 empty. Conformational changes of the P116 shown from the top view. Starting from the structure of the full P116 (open) a morphing is shown towards the structure of the empty P116. The speed of the movie may be adjusted in order to appreciate the conformational change.

Supplementary Video 11: Conformational change of P116 distal view

Morphing between the ribbon models from P116 and P116 empty. Conformational changes of the P116 shown from an arbitrary view. The movement of the four fingers (in

blue) towards the core domain (in orange) can be appreciated. The speed of the movie may be adjusted in order to appreciate the conformational change.

Supplementary Video 12: Conformational change of P116 distal view with ligands

Morphing between the ribbon models from P116 and P116 empty. Similar view as in movie 8, only the ligands (in red) present. In the closed conformation the 4 fingers (in blue) clash with the ligands.
