# Supplementary material for: Essential protein P116 extracts cholesterol and other indispensable lipids for Mycoplasmas
Source: Nat Struct Mol Biol. 2023 Feb 13;30(3):321–9. doi: 10.1038/s41594-023-00922-y (PMC10023570; doi:10.1038/s41594-023-00922-y)
Supplement: Supplementary file 2 — Reporting Summary [file 41594_2023_922_MOESM2_ESM.pdf]

nature portfolio

Corresponding author(s): Achilleas S. Frangakis

Last updated by author(s): Nov 30, 2022

Reporting Summary

Nature Portfolio wishes to improve the reproducibility of the work that we publish. This form provides structure for consistency and transparency in reporting. For further information on Nature Portfolio policies, see our Editorial Policies and the Editorial Policy Checklist.

Statistics

For all statistical analyses, confirm that the following items are present in the figure legend, table legend, main text, or Methods section.

n/a	Confirmed	
☐	☒	The exact sample size (*n*) for each experimental group/condition, given as a discrete number and unit of measurement
☐	☒	A statement on whether measurements were taken from distinct samples or whether the same sample was measured repeatedly
☒	☐	The statistical test(s) used AND whether they are one- or two-sided<br>*Only common tests should be described solely by name; describe more complex techniques in the Methods section.*
☒	☐	A description of all covariates tested
☒	☐	A description of any assumptions or corrections, such as tests of normality and adjustment for multiple comparisons
☒	☐	A full description of the statistical parameters including central tendency (e.g. means) or other basic estimates (e.g. regression coefficient) AND variation (e.g. standard deviation) or associated estimates of uncertainty (e.g. confidence intervals)
☒	☐	For null hypothesis testing, the test statistic (e.g. *F*, *t*, *r*) with confidence intervals, effect sizes, degrees of freedom and *P* value noted<br>*Give P values as exact values whenever suitable.*
☒	☐	For Bayesian analysis, information on the choice of priors and Markov chain Monte Carlo settings
☒	☐	For hierarchical and complex designs, identification of the appropriate level for tests and full reporting of outcomes
☒	☐	Estimates of effect sizes (e.g. Cohen's *d*, Pearson's *r*), indicating how they were calculated

Our web collection on statistics for biologists contains articles on many of the points above.

Software and code

Policy information about availability of computer code

Data collection	Freeware and commercial code has been used as referenced in the manuscript.<br>Electron microscopy: Serial EM v3.8, EPU 2.12<br>Light microscopy: ORCA-Fusion<br>Mass spectrometry: Compass 2.0
Data analysis	Freeware and commercial code has been used as referenced in the manuscript.<br>cryoSPARC v3.2.2, Artiatomi v0.1<br>LM: ImageJ<br>Mass spectrometry: MS dial lipidomics pipeline v 4.9, mTIC, R using the lipidr package, Frag-Pipe 18 using msfragger 3.5

For manuscripts utilizing custom algorithms or software that are central to the research but not yet described in published literature, software must be made available to editors and reviewers. We strongly encourage code deposition in a community repository (e.g. GitHub). See the Nature Portfolio guidelines for submitting code & software for further information.

Data

Policy information about availability of data

All manuscripts must include a data availability statement. This statement should provide the following information, where applicable:

- Accession codes, unique identifiers, or web links for publicly available datasets
- A description of any restrictions on data availability
- For clinical datasets or third party data, please ensure that the statement adheres to our policy

Cryo-electron microscopy densities of the original P116 density map (3.3 Å), the empty P116 (4 Å) and the refilled P116 (3.5 Å) have been deposited in the EM Data Base under the accession codes EMD-15274, EMD-15275 and EMD-15276, respectively. Model coordinates of original and empty P116 have been deposited in the PDB under the accession codes 8A9A and 8A9B, respectively. The mass spectrometry proteomics data have been deposited to the ProteomeXchange Consortium (http://proteomecentral.proteomexchange.org) via the PRIDE partner repository (Perez-Riverol et al, 2019) with the dataset identifier PXD037758.
Uniprot (P75556 Y213_MYCPN)

Human research participants

Policy information about studies involving human research participants and Sex and Gender in Research.

Reporting on sex and gender	out studies did not involve human research participants
Population characteristics	n/a
Recruitment	n/a
Ethics oversight	n/a

Note that full information on the approval of the study protocol must also be provided in the manuscript.

Field-specific reporting

Please select the one below that is the best fit for your research. If you are not sure, read the appropriate sections before making your selection.

☒ Life sciences ☐ Behavioural & social sciences ☐ Ecological, evolutionary & environmental sciences

For a reference copy of the document with all sections, see nature.com/documents/nr-reporting-summary-flat.pdf

Life sciences study design

All studies must disclose on these points even when the disclosure is negative.

Sample size	cryoEM:SupplementaryTable I. Sample size was chosen to such an amount in order to reach <4A resolution. Total particle size:<br>1.3 Mio particles for the purified P116<br>1.1 Mio particles for the empty P116<br>1.3 Mio particles for the refilled P116<br>58000 particle for the complex P116&HDL<br>Unsupervised classification was used to reject outliers.<br>LM: Five separate experiments for testing the antibodies and 3 experiments for testing the controls.<br>Counts of motile and non-motile mycoplasma cells were performed before adding antibodies. Once antibodies were added, the number of cells was determined after 10 min of incubation (table attached). The mean and standard deviation were computed from cell counts and no further statistical analyses were performed.
Data exclusions	cryoEM: All particles were included, classification was used to cluster the data set.<br>LM: No data was excluded<br>Mass-Spec: No data was excluded
Replication	cryoEM: Refinements and averages were done with random individual half sets. All experimental findings could be replicated.<br>LM: Five independent microcinematography replicates were made for each antibody and three replicates were performed for control experiments with no antibodies. All findings could be replicated.<br>3H Cholesterol Experiment: This experiment was performed once (n=1) as a proof of concept experiment to initiate the thorough analyses by LC-MS/MS.<br>LC-MS/MS: Proteomics and Lipidomics experiment was conducted as three independent replicates. All findings could be replicated.
Randomization	cryoEM: Single particle analysis and classification was done with CryoSparc. Refinements and averages were done with random individual half sets.

	LM: Field of view was randomly selected, all single cells were included in the analysis.<br>Mass-Spec: n/a
Blinding	cryoEM: Investigators were not blinded during grouping since it was computationally performed. HDL cholesterol transfer rate: Technician was blinded regarding the source of tritium-labeled HDL and P116 form.<br>LM: Investigators were not blinded.<br>Mass-Spec: n/a

Reporting for specific materials, systems and methods

We require information from authors about some types of materials, experimental systems and methods used in many studies. Here, indicate whether each material, system or method listed is relevant to your study. If you are not sure if a list item applies to your research, read the appropriate section before selecting a response.

Materials & experimental systems

n/a	Involved in the study
☐	☒ Antibodies
☒	☐ Eukaryotic cell lines
☒	☐ Palaeontology and archaeology
☐	☒ Animals and other organisms
☒	☐ Clinical data
☒	☐ Dual use research of concern

Methods

n/a	Involved in the study
☒	☐ ChIP-seq
☒	☐ Flow cytometry
☒	☐ MRI-based neuroimaging

Antibodies

Antibodies used	LM: Antibodies were generated within this work as described in the material and methods section.<br>Goat anti-mouse Alexa 555 (Invitrogen, Waltham, USA) secondary antibody: dilution: 1/2000;<br>P116 polyclonal antibodies: dilution 1/2000;<br>P1 polyclonal antibodies: dilution 1/2000;<br>Monoclonal antibodies: dilution 1/10: validation: indirect ELISA, western blot and immunofluorescence staining;<br><br>3H Experiment: A commercial anti-apoA-I-based assay is described in the material and methods section.
Validation	Supernatants from hybridoma cell lines derived from single fused cells were first investigated by indirect ELISA screening against the recombinant P116 ectodomain. Positive clones were also tested by Western blot against protein profiles from M. pneumoniae cell lysates and by immunofluorescence using whole, non-permeabilized M. pneumoniae cells (see below). Only those clones with supernatants revealing a single 116 kDa band in protein profiles and also exhibiting a consistent fluorescent staining of M. pneumoniae cells were selected and used in this work. ApoA1 was determined by an immunoturbidimetric assay, using a commercial kit, including standards for calibration and internal controls, adapted for a COBAS 6000 autoanalyzer (Roche Diagnostics, Rotkreuz, Switzerland).<br><br>LM: validation for Alexa 555: https://www.thermofisher.com/order/genome-database/dataSheetPdf?producttype=antibody&productsubtype=antibody_secondary&productId=A-21424&version=256; company names: Invitrogen; Cat Number: A21424.<br>P116 polyclonal antibodies: dilution 1/2000; validation: indirect ELISA, and Western blot; obtained in-house.<br>P1 polyclonal antibodies: dilution 1/2000; validation: indirect ELISA, and Western blot; obtained in-house.<br>Monoclonal antibodies: dilution 1/10: validation: indirect ELISA, western blot and immunofluorescence staining; obtained in-house.<br>Further validation is provided in the M&M section

Animals and other research organisms

Policy information about studies involving animals; ARRIVE guidelines recommended for reporting animal research, and Sex and Gender in Research

Laboratory animals	Mice:<br>- Two BALB/c females eight-week old were immunized to obtain polyclonal antibodies and monoclonal antibodies against P116.<br>- Two BALB/c females eight-week old were immunized to obtain polyclonal antibodies against P1.<br>The experimental procedures to immunize mice and obtaining monoclonal antibodies were approved by the Ethics Committee on Animal and Human Experimentation from the Universitat Autònoma de Barcelona by the document CEEAH 1002R3R2R.
Wild animals	No wild animals were used in the study.
Reporting on sex	Two BALB/c females eight-week old were used
Field-collected samples	no field collected samples were used in the study

Ethics oversight

The experimental procedures to immunize mice and obtaining monoclonal antibodies were approved by the Ethics Committee on Animal and Human Experimentation from the Universitat Autonòma de Barcelona by the document CEEAH 1002R3R2R. Also provided in the manuscipt in M&M section / timelapse

Note that full information on the approval of the study protocol must also be provided in the manuscript.
